# Make Mine Malaria

**DOI:** 10.4269/ajtmh.2012.12-0269

**Published:** 2012-07-01

**Authors:** Claire Panosian Dunavan

“Murugan sat suddenly upright, the sweat pounding off his face, not sure whether he was still dreaming or awake. The net was buzzing with mosquitoes; he could see them dancing like motes, in the finger of light that bisected his bed. His whole body was aflame, covered with bites. He had been scratching himself furiously in his sleep; he could see blood on his fingernails, and on the sheets.”

Sometime in the 1990s, I started writing occasional book reviews for The Los Angeles Times. The preceding passage from Amitav Ghosh's fantastical work—*“The Calcutta Chromosome: A novel of Fevers, Delirium & Discovery”*—launched my first-ever (and probably once-in-a-lifetime) review of a book inspired by a major milestone in tropical medicine. I loved Ghosh's historical-cum-near-future thriller featuring, among other characters: Antar, a depressed Egyptian émigré and computer specialist living in New York; Murugan, his “cocky little rooster” of a co-worker, and a self-proclaimed authority on Ronald Ross of the Indian Medical Service; and, later, Surgeon Major Ross himself. Of course, we all know Ross as the real-life “huntin' shootin' fishin' colonial type” (Ghosh's words, not mine) and researcher awarded a 1902 Nobel Prize for decrypting malaria's life cycle in mosquitoes.

Two other notable figures in *“The Calcutta Chromosome”* are Mangala, an illiterate (fictitious) sweeper-woman who—in the late 1890s—cleaned Ross's bungalow laboratory on the grounds of the Presidency General Hospital; and a man variously known as Lutchman, Lakshman, and Laakhan who was Ross's (true) patient and servant. In Ghosh's wickedly re-imagined version of history, it is silent Mangala who discovers malaria's inner life in mosquitoes, and then cleverly manipulates Ross into partial comprehension. Meanwhile, the low-caste cleaner is secretly treating sufferers of dementia paralytica (end-stage syphilis) with malaria-induced fever using avian parasites she harvests by slitting the throats of pigeons. Mangala ultimately perfects a genetic technique for transposing personality from one human being to another, thus assuring Lutchman's and her own immortality.

Revisiting this book, here is what I find most remarkable. Ghosh, a Bengali-born, Oxford- educated anthropologist-turned-lauded-global-author, fearlessly plaits real and imagined truth, fact and fantasy. Thus, his mesmerizing tale of science, technology, and culture also interrogates the colonial eco-system in which malaria's mysteries first began to unfold.

Today, the circumstances of Ronald Ross's great discovery remain somewhat murky. Was the once-diffident student really the lone genius portrayed in his memoirs published two decades after he (controversially) won the Nobel Prize? Or did his dogged quest benefit more than he cared to disclose from native helpers and their instinctual knowledge of malaria? Claire Chambers, a senior lecturer in post-colonial literatures at the University of Leeds, speculates that “Ross's high-handed treatment of his Indian patients and servants” is one reason Amitav Ghosh was inspired (or angered) to write the chimerical *“Calcutta Chromosome”* in the first place.

Fast-forward 15 years and you'll find another malaria visionary of sorts in Dr. Annick Swenson, the tyrannical, rogue researcher who dominates *“State of Wonder,”* Ann Patchett's latest work of fiction. This novel opens in Minnesota but soon hurtles its junior protagonist, Dr. Marina Singh, to Manaus, Brazil, then up a tributary of the Rio Negro to Swenson's hidden research station. Why hidden? Although bankrolled by the pharmaceutical giant Vogel, the irascible, elusive Swenson has not yet revealed the source of a local nostrum that allows females of the Lakashi Amazon tribe to bear children over their entire, adult lifespan. Nor has she even hinted to her corporate bosses back home that the offspring of these perpetually fertile Amerindians are resistant to malaria.

Swenson has her reasons for failing to share the anti-malarial magic of the forest “medicine” whose bio-activity stems from an admixture of living tree bark, moth eggs, and human saliva. As she tells Singh toward the end of the book: “When we get one drug, we'll have the other, and I don't see the harm in making an American pharmaceutical company pay for a vaccination that will have enormous benefits to world health and no financial benefits for company shareholders. The people who need a malaria vaccine will never have the means to pay for it. At the same time I will give them a drug that will, if anything, undermine the health of women and make them a truly obscene fortune. Isn't that a reasonable exchange?”

Patchett fans often cite *“Bel Canto”*—the author's luminous 2001 novel about a botched guerilla capture of an international diva and her fans—as her chef d'oeuvre. Whereas *“State of Wonder”* is a jungle page-turner, *“Bel Canto”* is a breath-taking work of modern literature.

On the other hand, *“State of Wonder”* offers up dark mefloquine dreams, mysterious fevers, an endearing deaf-mute named Easter, a pair of daft eco-groupies, a gala evening at the Teatro Amazonas, poison arrows, deadly snakes, rumors of cannibals, riverbank torches, ululations, and flawed, complex characters in a story laced with equally-complex bio-ethics. In the end, I found it irresistible.

Now, for a stand-out in the recent canon of tropical non-fiction. Sonia Shah's *“The Fever: How Malaria has Ruled Humankind for 500,000 Years”* is the latest in a decade-long bounty of popular books and works of journalism about malaria—and the most ambitious. However first, kudos on style. Shah's flair for conversational, dramatic narrative compels from page one. There the author starts by recalling childhood visits to southern India and her early terror of mosquito-borne disease.

“While my cousins snore on the bed mats laid across the floor beside me, glistening bodies bathed in the warm night breeze, my sleeping mat is ensconced in a hot, gauzy cage. The mosquitoes descend from the darkened corners of the whitewashed room and perch menacingly on the taut netting, ready to exploit any flicker of movement from their prey within.”

The New England native, we soon learn, is further constrained by her family's Jain creed prohibiting all violent acts, whether they involve eating meat, swatting a fly, walking on grass—or crushing a dapple-winged Diptera.

Fast forward 30 years, and Shah has arrived in Latin America's northernmost *Plasmodium falciparum* outpost accompanied by Jose Calzada of the Gorgas Memorial Institute. In 2005, when Calzada and co-workers first met the native Kuna of the jungle hamlet of Chepo, Panama, Shah relates, “nearly half of the settlement was fevered, terrified, immobilized in their hammocks.” A few paragraphs later, Shah visits another familiar falciparum stronghold in Malawi. There, she is greeted by *ASTMH* member Terrie Taylor, deftly captured in this passage:

“In her fifties, Taylor wears long, loose skirts and keeps her frizzy, brown hair parted in the middle. She starts talking straightaway, as if we've known each other for years, grabbing my shoulder and making gently irreverent cracks. She marches through the airport waving and calling out greetings to nearly everyone we pass. The air in Blantyre, as we exit the airport, is scorching and heavy with humidity. Soon the rains will start, and the public hospital where Taylor works will be full of frightened parents proffering their limp, fevered children.”

Of her ultimate destination, Shah writes: “The pediatric research ward, at the very edge of the hospital complex, consists of two large rooms holding about fifteen wooden raised beds each, a narrow fluorescent-tube-lit hallway, and some barren, closet-size offices, including Terrie Taylor's. Unlike the rest of the hospital, with its crowds and smells, the research ward has certain serenity to it, despite the drumbeat of child deaths that occur within its walls. Most of the young patients here are deathly ill with malaria, and comatose. There is no welter of plastic tubing or beeping machines around them as one would see in the West. Their small bodies rest on the high beds unadorned. They appear to be simply asleep.”

Shah's gifted, evocative story-telling partnered with deep research yields a dense, layered, yet well-paced text embracing history, biology, ecology, economics, and past and present policy battles. Although *“The Fever”* could prove a challenging read for malaria rookies, its author has certainly raised the bar in presenting the complex calculus of the ancient scourge. At the same time, however, she balks at forecasting gains from today's scaled-up control and elimination efforts. Despite her compassion for sufferers, Shah remains cynical, (in the words of one reviewer: taking “no prisoners, blasting everyone …”), it seems, about the modern malaria enterprise.

Is *“The Fever*'s*”* acerbic tone mere journalistic device or does it reflect true doubt and dismay over the still-unfolding drama of man versus malaria? I wondered after breathing in its final page. Someday, Sonia Shah must come to a meeting of the American Society of Tropical Medicine and Hygiene and tell all.

## Work Cited

Chambers C, 2003. Postcolonial science fiction: Amitav Ghosh's The Calcutta Chromosome. *The Journal of Commonwealth Literature 38:* 58–72.

Ghosh A, 1995. *The Calcutta Chromosome: A Novel of Fevers, Delirium & Discovery*. New York: Avon Books.

Patchett A, 2011. *State of Wonder*. New York: Harper Collins.

Shah S, 2010. *The Fever: How Malaria Has Ruled Humankind for 500,000 Years*. New York: Farrar, Straus and Giroux.

